# Excited-State Absorption Drives Low-Energy Optical Limiting in Oligothiophenes

**DOI:** 10.1021/acs.jpclett.5c02340

**Published:** 2025-11-06

**Authors:** Mustapha Driouech, Michele Guerrini, Caterina Cocchi

**Affiliations:** † Friedrich-Schiller Universität Jena, Institute for Condensed Matter Theory and Optics, 07743 Jena, Germany; ‡ 232751 Carl von Ossietzky Universität Oldenburg, Institute of Physics, 26129 Oldenburg, Germany; § Center for Nanoscale Dynamics (CeNaD), 26129 Oldenburg, Germany

## Abstract

Optical limiting (OL), a crucial mechanism for protecting human eyes and sensitive sensors from intense radiation, relies on understanding the optical nonlinearities acting on the systems. Assessing and disentangling the effects at play is crucial to predict and control the nonlinear optical response in real materials. In this *ab initio* study based on real-time time-dependent density-functional theory, we investigate nonperturbatively the absorption spectra of a set of thiophene oligomers, the building blocks of technologically relevant organic semiconductors, excited by broadband radiation of increasing intensity. Under strong electric fields, the absorption cross section grows significantly below the onset of linear excitations, exhibiting saturation typical of OL. By exciting the oligothiophenes with a train of pulses targeting the first and second excited states of each moiety and analyzing the resulting population dynamics, we reveal excited-state absorption (ESA) in the near-infrared to visible region. Our results indicate ESA as the driving mechanism for OL in oligothiophene molecules, thereby providing important insight into the design of novel compounds with optimized nonlinear optical characteristics.

## Graphical abstract

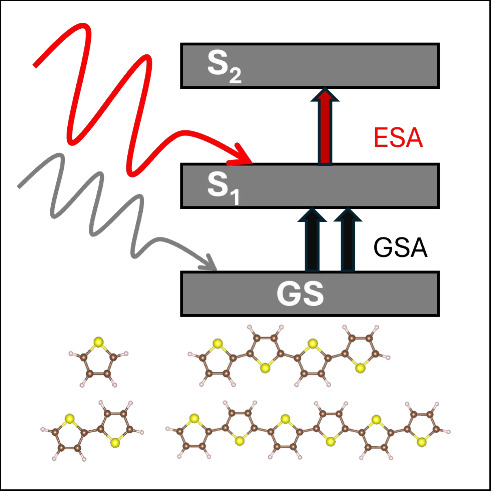

Optical limiting (OL), the ability of a material to attenuate intense light transmission, is of critical importance for laser safety technologies,^1,2^ including the protection of sensitive optical sensors^3^ and human eyes.^4–6^ Designing efficient OL materials hinges on a fundamental understanding of the underlying optical nonlinearities. While mechanisms like reverse saturable absorption, excited-state absorption (ESA), and two-photon absorption are recognized as primary drivers of OL,^7^ identifying and disentangling their contributions in specific material classes remains a challenging task. Solving this conundrum is essential for advancing OL-based technologies.

Carbon-conjugated molecules have been intensively studied for OL due to their extended electronic π-network, large polarizability, and inherent chemical flexibility.^8^ Porphyrins and phthalocyanines have received significant attention, owing to their broad transparency window in the visible region that can be populated by intense radiation.^9,10^ While this interest has significantly promoted the study of OL and the development of related applications,^11^ it has diverted attention from other potentially relevant compounds, such as oligothiophene molecules. Their rich spectrum of linear excitations together with their tunability via chemical functionalization and length modulation^12–14^ have established them as building blocks for organic electronic devices.^15–18^ Although recent experimental studies on oligothiophene-functionalized graphene^19,20^ indicate their favorability for nonlinear optics and OL, the potential of oligothiophenes in these relevant technological areas remains largely unexplored. A detailed investigation of their response to strong fields is urgently needed to assess their ability as efficient OL compounds.

*Ab initio* methods are particularly well-suited for studying nonlinear optical properties of molecules. In contrast to empirical models, they do not require any input from experiments, thus representing a reliable and predictive tool to characterize new compounds. Real-time time-dependent density functional theory (RT-TDDFT), a nonperturbative first-principles approach, offers a particularly versatile framework.^21^ The “*δ*-kick” method introduced by Yabana and Bertsch to simulate linear absorption spectra^22^ and subsequently extended to probe nonlinear excitations driven by intense broadband radiation,^23–25^ is an effective tool to access optical nonlinearities of molecules. This approach leverages the intrinsic nonperturbative nature of RT-TDDFT, capturing nonlinear effects of any order without cumbersome and numerically costly expansions of the response function at given orders of perturbation. An effective combination of RT-TDDFT and linear-response time-dependent density functional theory was proposed by Fischer et al. to access ESA.^26^ Efficient schemes for pump–probe^27,28^ and multidimensional spectroscopy^29^ are implemented in RT-TDDFT through the inclusion of pulsed electric fields of tunable shape, frequency, duration, and polarization, evolving with the system during a femtosecond (fs) time window.^27^ This setup enables exploring dynamical charge transfer and nonequilibrium dynamics in organic, inorganic, and hybrid materials,^30–38^ including the influence of vibronic couplings when combined with Ehrenfest dynamics.^39–42^

In this work, we apply the δ-kick and pump–probe schemes of RT-TDDFT to investigate OL in four thiophene oligomers composed of 1, 2, 4, and 6 rings, adopting the approach introduced in ref 28. and complementing the method designed by Fischer et al.^26^ The choice of this set of three even-numbered oligomers supplemented by the single thiophene ring is motivated by existing theoretical and experimental literature,^43,44^ reporting strong evidence for loss of planarity above four rings^43^ and indicating six rings as representative for the effective conjugation length of larger thiophene segments.^44^ By exciting the selected molecules with broadband radiation of increasing intensity polarized along all Cartesian directions, we find enhanced nonlinear absorption in the near-infrared to visible region below the onset of the linear spectrum. The saturation of this band upon increasing field intensity confirms its relation to OL.

By impinging the oligothiophenes with a train of fs pulses in resonance with the lowest energy excitation polarized along the long molecular axis, and analyzing the resulting population dynamics, we rationalize their nonlinear optical behavior in terms of ESA. We perform our study within the adiabatic local density approximation (ALDA), which provides an optimal trade-off between qualitative accuracy and computational costs and stability. Despite the known limitations of this method in predicting the full excited-state manifold in organic systems,^45–52^ including the exact ordering and composition of higher excited states, ALDA is sufficiently robust for the qualitative analysis, performed in a nonperturbative framework, presented in this work. Our results have two important implications: they disclose the potential of oligothiophenes for OL in the near-infrared to visible region, depending on their length, and confirm the ability of RT-TDDFT to shed light on optical nonlinearities of conjugated molecules in an insightful and yet computationally efficient way.

We start our analysis by computing the absorption spectra of the four considered oligothiophenes applying δ-kicks of increasing intensity, ranging from a weak perturbation delivering the linear spectrum^22^ to strong fields triggering pronounced nonlinear response.^23^ The linear spectrum of the single thiophene ring (1T), obtained with a δ-kick of magnitude 0.001 Å^–1^, exhibits a peak around 5.5 eV (Figure 1a), which, due to intrinsic broadening (details in the Computational section) encompasses the two lowest-energy excitations polarized in the *y*- and *x*-direction, respectively^53^ (see Table S1 for the perturbative analysis of the linear excitations). Our findings are in very good agreement with quantum chemistry predictions^54,55^ and experimental results.^56,57^ Increasing the perturbation strength by 2 orders of magnitude (δ-kick κ = 0.1 Å^–1^) preserves the first absorption peak around 5.5 eV, but also leads to a nonzero cross section at low energies, between 0.5 and 2.5 eV. Larger values of the δ-kick induce a blue shift in the peak associated with the first linear excitation around 5.5 eV, a consequence of memory effects being neglected in the adopted adiabatic local density approximation.^58,59^ Importantly, the low-energy absorption band grows in intensity and further extends in energy with the magnitude of the kick, merging with the peak at 5.5 eV for κ ≥ 0.8 Å^–1^ (Figure 1a).

**Figure 1:**
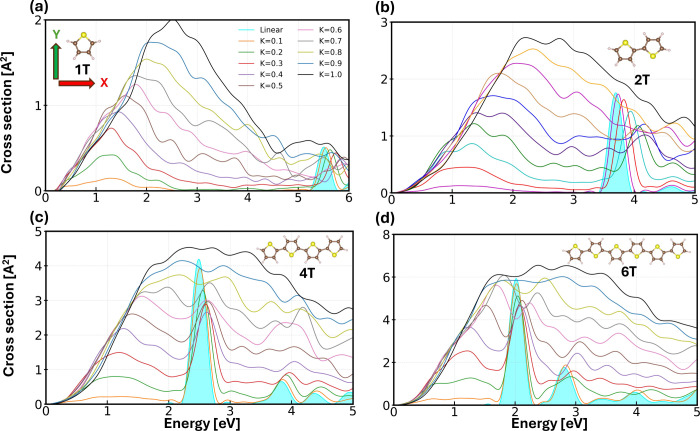
Absorption cross section, computed as the trace over the three Cartesian components, of a) 1T, b) 2T, c) 4T, and d) 6T excited by instantaneous, broadband excitations of increasing intensities polarized along all Cartesian directions. Linear absorption spectra are obtained with a δ-kick of 0.001 Å^–1^ (cyan filled areas), while nonlinear spectra are excited by kicks (κ in Å^–1^) specified in the legend of panel a). Insets: Ball-and-stick representations of the investigated oligothiophenes with C atoms depicted in brown, S atoms in yellow, and H atoms in pink. The Cartesian coordinate system in panel a) is referred to all molecules.

A similar behavior is exhibited by bithiophene (2T) under analogous excitation conditions. A weak δ-kick κ = 0.001 Å^–1^ leads to the linear absorption spectrum of this molecule, characterized by a sharp resonance around 3.8 eV (Figure 1b), in overall agreement with our linear-response calculations (Table S2), experiments^57,60^ and quantum-chemistry calculations.^61^ Increasing the field intensity induces again a slight blue shift of the main absorption maximum due to the lack of memory effects in our calculations,^58,59^ and the appearance of a broad absorption band centered at 1 eV (Figure 1b). An even stronger perturbation shifts the maximum to 4 eV and further extends the energy range of the low-energy band across the entire visible range.

The linear absorption spectra of quaterthiophene (4T) and sexithiophene (6T) are characterized by strong resonances in the visible region, centered at approximately 2.5 eV (Figure 1c) and 2 eV (Figure 1d), respectively. These findings are in line with our linear-response calculations (Tables S3 and S4, respectively), previous *ab initio* calculations^62,63^ and experiments.^64–66^ In the spectrum of 6T, a second weaker maximum appears at 2.8 eV. For 4T, this excitation has a lower oscillator strength and a higher energy, appearing at approximately 4 eV. In analogy with the shorter oligomers, perturbing these molecules with δ-kicks of increasing intensity promotes absorption in the low-energy spectral region, corresponding to infrared frequencies. The first absorption peak in the linear regime remains well-defined up to κ = 0.4 Å^–1^, despite losing oscillator strength and being slightly blue-shifted (Figure 1c,d). In the spectrum of 4T, larger δ-kick intensities, up to κ = 0.7 Å^–1^ enhance the spectral strength of this resonance while broadening it, while for κ > 0.7 Å^–1^, a continuous absorption band rising at approximately 0.5 eV up to 4 eV is formed (Figure 1c). For 6T, the situation is more faceted. Kick strengths between 0.6 Å^–1^ and 0.8 Å^–1^ generate a two-peak structure in the absorption spectrum (Figure 1d), while, similar to 4T, stronger intensities give rise to featureless absorption from infrared to near-UV frequencies. The second absorption peak in the linear regime is more sensitive to the kick strength in the spectra of both molecules, where it is no longer distinguishable from κ = 0.5 Å^–1^ in 4T (Figure 1c) and κ = 0.6 Å^–1^ in 6T (Figure 1d).

It is worth mentioning that the low-lying triplet excitations of the considered thiophene oligomers lie in the region where the signatures of ESA below the onset of linear absorption appear.^66,67^ These additional channels can contribute to the nonlinear response of the molecules excited by strong electric fields, as suggested by Isborn and Li.^68^ However, spin-mechanisms can be rigorously captured only within a spin-unrestricted framework, like the one adopted in ref 68. In the spin-restricted RT-TDDFT method adopted in this work, the spin operator is constrained to Δ*S* = 0, and triplet states cannot be accessed even under intense irradiation. As such, we are unable to comment further on the role of single-triplet transitions in OL, and we reserve for future work dedicated theoretical and computational efforts for an in-depth analysis of this crucial physical process.

The large low-energy absorption cross-section in the nonlinear spectra of the considered thiophene oligomers suggests the emergence of OL, further confirmed by its saturation upon integration in the relevant energy window (Figure S1). In contrast to phthalocyanine, where the spectral window populated by intense, broadband radiation is identified between the two main absorption bands^23^ hosting dark transitions, enhanced nonlinear absorption in the spectra of oligothiophenes appears below the lowest-energy excitation in the linear regime. This finding suggests that ESA drives the response of these molecules to intense broadband radiation. To test this hypothesis, we perform an additional set of RT-TDDFT simulations, exciting the molecules with time-dependent pulses. By targeting the lowest-energy excitation (S_0_ → S_1_, see Tables S1–S4 in the Supporting Information and the Computational Section below), we drive the molecules out of the linear regime. Next, we probe the population dynamics by impinging the moieties with a second pulse, delayed by 15 fs with respect to the first one and with carrier frequency in resonance with the low-energy absorption band emerging in the nonlinear regime.

The differential absorption spectra computed for each molecule after the application of the first pulse targeting S_0_ → S_1_ are dominated by the excitation in resonance with the carrier frequency of the applied pulse (dashed vertical line in Figure 2). In addition, a weak but distinct absorption peak appears in the low-energy region of each spectrum, in the same window where strong δ-kicks give rise to nonzero absorption cross section (Figure 1). For 1T, this maximum appears in the visible region at 2.7 eV (Figure 2a), while for the longer oligomers, it is found at infrared frequencies, around 1 eV in 2T (Figure 2b), and close to 0.5 eV for both 4T (Figure 2c) and 6T (Figure 2d). The differential absorption spectra of the two longest molecules, 4T and 6T, are characterized by many more features compared to those of the shorter moieties. This is due to the higher density of excited states, which are involved in the nonlinear excitation. Nonetheless, all nonlinear spectra exhibit the same key characteristic, namely an absorption band in the near-infrared region, which is compatible with ESA.

**Figure 2:**
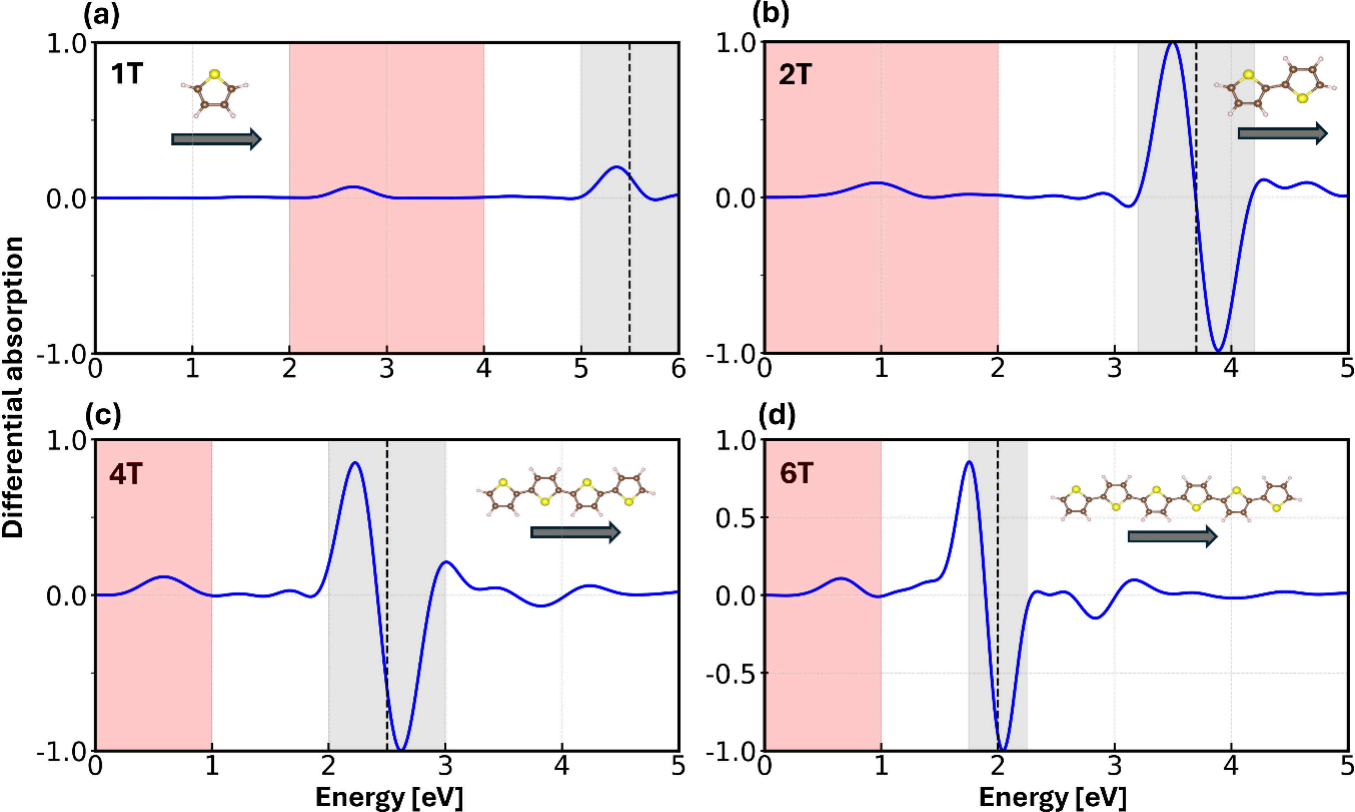
Differential absorption spectra of a) 1T, b) 2T, c) 4T, and d) 6T excited by a Gaussian pulse with peak intensity *I* = 50 GW/cm^2^, polarization oriented along the long molecular axis as indicated by the dark gray arrows in the insets, and photon energy of a) 5.5 eV, b) 3.7 eV, c) 2.5 eV, and d) 2.0 eV, in resonance with the first excitation in the linear spectra of the oligomers (dashed bars). The gray area indicates the bandwidth of the applied pulse, while the red shaded region highlights the low-energy nonlinear absorption band subsequently probed by the second pulse to access population dynamics.

To confirm this hypothesis, we monitor the electronic population dynamics by estimating the number of excited electrons through the contributions from time-dependent single-particle states ϕ_*j*_(*t*), see Computational Section below, projected onto their ground-state counterparts at *t* = 0:^28^

$$N_{\mathrm{ex}}\left(t\right)=2\overset{\mathrm{unocc}}{\underset{m}{\sum }}\overset{\mathrm{occ}}{\underset{j}{\sum }}{\left|\left\langle \phi_{m}\left(0\right)\right|\phi_{j}\left(t\right)\rangle \right|}^{2}$$ 1

where the prefactor 2 accounts for spin degeneracy and the sum over the unoccupied states runs up to 100, covering an energy range of approximately 25 eV above the LUMO of all oligomers. We perform this analysis in two steps. Upon the application of the first pulse (gray area in Figure 3), which is set in resonance with the S_0_ → S_1_ excitation in each molecule (gray area in Figure 2), we evaluate the amount of charge depleted from the ground state and promoted to the first excited state. In the single-particle framework provided by RT-TDDFT, we compute these contributions in terms of the occupation of the highest-occupied molecular orbital (HOMO) and the lowest-unoccupied molecular orbital (LUMO) involved in this transition, see Tables S1–S4. Next, we apply a second pulse targeting the low-energy absorption maximum (red area in Figure 3) and estimate the number of electrons promoted from S_1_ to higher excited states S_n_, including contributions from LUMO+1 up to LUMO+10, see Figure S2.

**Figure 3:**
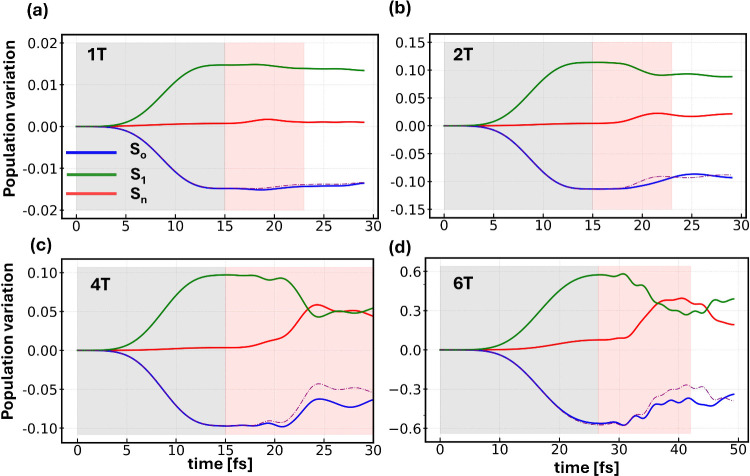
Variation in time of the population of the ground state (S_0_) as well as of the first (S_1_) and higher (single) excited states (S_n_) of a) 1T, b) 2T, c) 4T, and d) 6T, driven by a train of two Gaussian pulses in resonance with the S_0_ → S_1_ excitation (gray area) and with the absorption maximum below the linear onset (red area). The dotted-dashed purple line indicates the inverse of the population variation of S_1_.

The results visualized in Figure 3 validate our hypothesis regarding the role of ESA in driving low-energy nonlinear absorption in the oligothiophene molecules excited by strong broadband radiation (see Figure S2 for the detailed contributions of involved unoccupied molecular orbitals). In 1T, the application of the first pulse with a carrier frequency of 5.5 eV triggers the occupation of S_1_ at the expense of S_0_ (Figure 3a, gray area). After 15 fs, when the second pulse with a carrier frequency of 3 eV is activated (Figure 3a, red area), we notice a slight reduction in S_1_ due to the population of higher excited states (red curve). This is a signature of ESA. Concomitantly, the ground-state population rises, but without perfectly mirroring the S_1_ population (dashed-dotted purple curve in Figure 3a), as during the action of the first pulse. We attribute this behavior to ground-state bleaching during the population of excited states beyond S_1_.

In 2T, the mechanisms described above for 1T are amplified both quantitatively (compare the *y*-axis scale in Figure 3a and Figure 3b) and qualitatively. The first pulse with a carrier frequency of 3.7 eV shifts more than 0.1 *e* from the ground state to S_1_. After 20 fs, when the second pulse has reached its peak, the population of the first excitation decreases at the advantage of higher states (Figure 3b, red area). Again, signatures of a slight ground-state bleaching are visible through the mismatch between the blue solid curve and the dashed-dotted purple line in Figure 3b.

The situation is more faceted for 4T (Figure 3c). Here, the activation of the second pulse leads to a sizable depletion of S_1_, which becomes less occupied than the other excited states after the second pulse has reached its peak. The S_n_ population receives non-negligible contributions also from the ground state, as indicated by the blue curve departing from the purple one, representing the inverse population of S_1_. In 6T, occupation variations become even larger in magnitude (Figure 3d) with S_1_ losing more than 50% of the population gained from the first pulse during irradiation with the second. Higher excited states take up more than 30% of the total electronic population upon the application of the second pulse, with ground-state bleaching contributing to the process. It is worth noting that for 6T, excited states beyond S_1_ are populated already by the first pulse (Figure 3d, gray area). The more elaborate composition of the first excited state in this long oligomer (see Table S4) likely plays a role here. However, we cannot exclude additional nonlinear effects emerging in the laser-driven dynamics, which will be investigated in follow-up work. A full account of these effects also includes higher-order contributions, e.g., quadrupolar terms, as well as the influence of nuclear motion related to polaron formation.^46,69–72^ Both aspects will be specifically addressed in upcoming dedicated studies.

In summary, our RT-TDDFT simulations unambiguously attribute to ESA the absorption below the linear onset induced by intense, broadband radiation in the spectra of 1T, 2T, 4T, and 6T. The different symmetry of the even-numbered oligomers with respect to 1T confirms the generality of this effect, in agreement with existing knowledge on the nonlinear response of oligothiophenes.^73^ The appearance of ESA is associated with OL and discloses the potential of these molecules to be employed as active components for corresponding applications working in the near-infrared to visible region. We revealed this mechanism by exciting the systems with a train of two pulses tuned in resonance with the first linear excitation in each molecule (S_0_ → S_1_) and with transitions from the S_1_ to higher excited states. The resulting electronic population dynamics support this interpretation, revealing ground-state depletion and the subsequent occupation of the first excited state under the action of the first pulse, as well as the population of higher excited states when the second pulse is turned on. While the length and symmetry of the molecules and the consequent electronic-structure variations induce expected quantitative changes, as discussed in the literature,^73,74^ the same qualitative behavior persists in the entire series, confirming ESA as the key mechanism driving OL in these compounds.

In conclusion, this study reveals the significant potential of oligothiophenes as OL compounds, featuring an active region in the near-infrared to visible band that effectively complements the window covered by more established carbon-conjugated molecules like phthalocyanines and porphyrins. Our detailed RT-TDDFT analysis based on a computational framework complementary to the one proposed by Fischer et al. combining linear-response and real-time TDDFT,^26^ identifies ESA as the main driver of OL in oligothiophenes. Our results not only highlight the capability of RT-TDDFT as a parameter-free, nonperturbative *ab initio* method to efficiently simulate and unveil the fundamental origins of optical nonlinearities in conjugated molecules but also set the stage for the rational design of optimized OL compounds with in-depth insight into the underlying physical mechanisms. Deeper and more quantitative spectral predictions can be achieved by adopting hybrid functionals, and further physical insight can be obtained by including multipolar contributions and triplet channels to the nonlinear cross section. As such, this work should not be regarded as an arrival point but rather as the first stage of an intensive research on oligothiophenes as promising materials for optical limiting.

## Computational Methods

### Theoretical Background

The calculations presented in this work are based on RT-TDDFT, based on the time propagation of the time-dependent Kohn–Sham (TDKS),

$$i\hbar \frac{\partial }{\partial t}\psi_{i}\left(r,t\right)=\left(-\frac{\hbar^{2}}{2m}\nabla^{2}+V_{\mathrm{eff}}\left(r,t\right)\right)\psi_{i}\left(r,t\right)$$ 2

where ψ_*i*_(**r**, *t*) are the TDKS states and *V*_eff_(**r**, *t*) is the time-dependent effective potential, including the contributions from the external potential, accounting for electron–nuclear interactions, the Hartree potential, and the exchange-correlation potential. The time-dependent electron density, computed from the solution of the TDKS as ρ­(**r**, *t*) = ∑ _*i*_^*occ*^|ψ_*i*_(**r**, *t*)|^2^, enters the expression of the time-dependent dipole moment, which in the *x*-direction reads:

⟨x(t)⟩=∫xρ(r,t)dr 3

The Fourier transform of eq 3,

$$\left\langle \mathrm{x̃}\left(\omega \right)\right\rangle =\int \left\langle x\left(t\right)\right\rangle e^{i\omega t}dt$$ 4

is proportional to the polarizability, whose imaginary part enters the expression of the absorption cross section.^22^

To compute the nonlinear response, we employed two computational approaches. In the so-called δ-kick scheme, introduced by Yabana and Bertsch^22^ and later applied by Cocchi et al. to simulate OL,^23^ the system is excited by an instantaneous broadband electric field, causing the electronic wave functions to acquire a phase factor expressed as ψ_*i*_(**r**, *t*) → ψ_*i*_(**r**, *t*)*e* ^*iκ*·*x*^ in the length gauge. The associated electric field is

$$E\left(t\right)=E_{0}\delta \left(t\right)$$ 5

with the amplitude $E_{0}=\hbar \kappa /e$ depending linearly on the kick strength κ. The molecules are also excited with a time-dependent Gaussian-enveloped electric field of the form

$$E\left(t\right)=E_{0}\exp\left[-\frac{{\left(t-t_{0}\right)}^{2}}{2\tau^{2}}\right]\cos\left[\omega_{0}(t-t_{0})\right]$$ 6

where ω_0_ is the carrier frequency, *t*_0_ the pulse center time, and τ the standard deviation related to the full-width half-maximum of the Gaussian pulse indicated as the bandwidth of the laser indicated by the gray area in Figure 2.

### Computational Details

All calculations reported in this work were performed with the code Octopus,^75^ implementing RT-TDDFT on real-space numerical grids. The molecular geometries adopted in this study were optimized using the FIRE algorithm^76^ with a threshold of 10^–4^ eV/Å for the residual interatomic forces. These simulations were performed within the local-density approximation (LDA, Perdew–Zunger functional^77^) using the Hartwigsen-Goedecker-Hutter pseudopotentials in a minimum box with a radius of 6 Å, and a real-space grid spacing of 0.18 Å.

For the time-propagations triggered by the δ-kick,^22^ we adopted Troullier-Martins pseudopotentials^78^ and the adiabatic LDA,^77^ which ensures superior numerical stability at low computational costs. In these runs, the real-space box size was set to 15 Å and the grid spacing to 0.18 Å. These values were carefully chosen to avoid spurious effects under strong-field irradiation. No absorbing boundaries are included. The TDKS equations were propagated using the enforced time-reversal symmetry propagator and Lanczos algorithm^79^ with a time step of 10^–3^ fs = 1 as for a total duration of 15 fs, giving rise to an intrinsic broadening of 44 meV in the linear absorption spectra. The duration of the time-step was checked to represent the optimal trade-off between computational costs and numerical stability (Figures S3–S6). The adopted δ-kicks were polarized in all three Cartesian directions to capture the full spectral response of the molecules. The electronic contributions to each linear excitation were resolved using the Casida method^80^ (Tables S1–S4).

We tested the validity of the adiabatic LDA by benchmarking the linear absorption spectrum computed from RT-TDDFT with the kick method^22^ and in linear response (Casida approach^80^) against CAM-B3LYP.^81^ This range-separated hybrid functional is considered the state-of-the-art method for describing optical excitations in conjugated molecules,^45^ including thiophenes.^46–52^ This comparison (Figure S4) reveals the expected rigid red-shift of the LDA excitation energies compared to CAM-B3LYP results. A deeper analysis of the lowest-energy excitations (see Figure S7 and compare Tables S5–S12 with Tables S1–S4) shows a good agreement in the composition and relative oscillator strength of the first bright excitation, matching available experimental data^82^ recorded at room temperature in solution (Table S13). Since the focus of this work is qualitative and aimed at establishing ESA as the main driver for OL in oligothiophenes, the accurate description of S_1_ by ALDA is the key point to support our conclusions, even though higher-energy excited states may appear in switched order and include different orbital contributions compared to benchmarks based on hybrid functionals. For example, a pronounced functional sensitivity can manifest itself in the calculation of the ESA cross sections,^26^ which is, however, not within the scope of this work. Despite its limitations, the numerical stability and efficiency ensured by ALDA in comparison with range-separated hybrid functionals are crucial for performing such a comprehensive, nonperturbative analysis across a series of large oligomers of technological relevance.

The runs performed with the Gaussian-shaped electric fields were carried out in a spherical box with a radius of 5 Å and grid spacing of 0.25 Å. The time-step is set to 2.9 as. To calculate the differential absorption (Figure 2), we excited 1T, 2T, and 4T with a pulse of peak intensity *I* = 50 GW/cm^2^, while for 6T we took *I* = 10 GW/cm^2^. The carrier frequencies were set in resonance with the S_0_ → S_1_ transition of each molecule, namely 5.5 eV for 1T, 3.7 eV for 2T, 2.5 eV for 4T, and 2 eV for 6T. To amplify variations in the population dynamics (Figure 3), we applied the second pulse with a peak intensity of 2 TW/cm^2^ for 1T and 2T, 1 TW/cm^2^ for 4T, and 500 GW/cm^2^ for 6T. The carrier frequency was set to 3, 1, 0.5, and 0.5 eV for 1T, 2T, 4T, and 6T, respectively, targeting the absorption peak emerging in the differential absorption displayed in Figure 2. The lower pulse adopted for 6T compared to the shorter oligomers is due to the length of this molecule. To selectively excite the HOMO→LUMO transition in 6T, a narrower pulse of 0.5 eV is necessary, corresponding to different pulse timing compared to 1T, 2T, and 4T.

## Supplementary Material



## Data Availability

The data generated in this study, including the corresponding input files, are available free of charge on Zenodo, DOI: 10.5281/zenodo.16037460.

## Notes

Integrated cross sections of the thiophene oligomers, assessment of the numerical stability and accuracy of our spectra obtained with strong δ-kicks, Casida analysis of their linear excitations, and molecular orbital contributions to the population analysis (PDF)
