# Supplementary material for: Excited-State Absorption Drives Low-Energy Optical Limiting in Oligothiophenes
Source: J Phys Chem Lett. 2025 Nov 6;16(45):11867–75. doi: 10.1021/acs.jpclett.5c02340 (PMC12621244; doi:10.1021/acs.jpclett.5c02340)
Supplement: Supplementary file 1 [file jz5c02340_si_001.pdf]

SUPPORTING INFORMATION

Excited State Absorption Drives Low-Energy Optical Limiting in Oligothiophenes

Mustapha Driouech,[†,‡] Michele Guerrini,[‡] and Caterina Cocchi[*,†,‡,¶]

†*Friedrich-Schiller Universität Jena, Institute for Condensed Matter Theory and Optics, 07743 Jena, Germany*

‡*Carl von Ossietzky Universität Oldenburg, Institute of Physics, 26129 Oldenburg, Germany*

¶*Center for Nanoscale Dynamics (CeNaD), 26129 Oldenburg, Germany*

E-mail: caterina.cocchi@uni-jena.de

Integrated Cross Sections

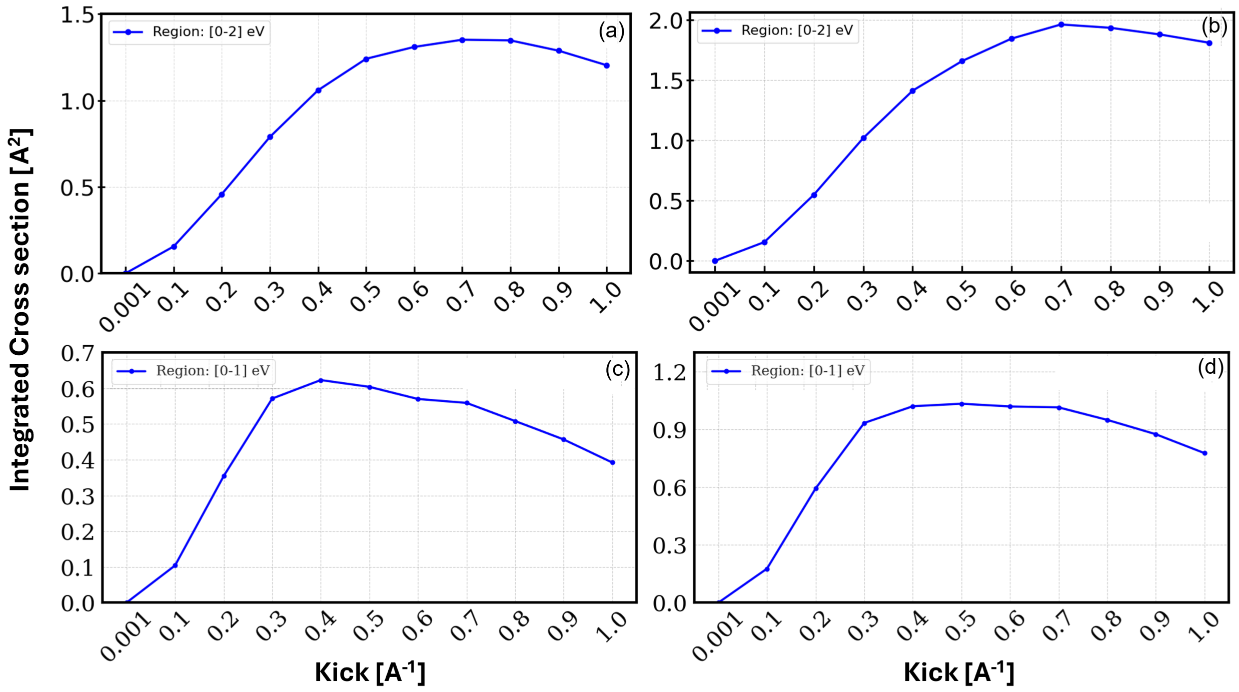

Figure S1: Integrated cross section for a) 1T, b) 2T, c) 4T, and d) 6T in the region indicated in the legends, namely [0.0 – 2.0] eV for 1T and 2T, and [0.0 – 1.0] eV for 4T and 6T.

Casida Analysis

In the following, we display the characteristics of the first five excitations computed with Octopus using the Casida scheme for linear response (LDA functional). We checked that the first excitation predicted by Casida in the spectrum of 1T is a spurious effect of the local exchange-correlation functional and of the numerical grids implemented in Octopus (compare Tables S5 and S6). Due to its extremely weak oscillator strength, we can exclude any influence of this feature on the results of this work. A comparison with the spectra computed from RT-TDDFT in the linear regime (kick strength 0.001 $Å^{-1}$) is shown in Figure S4.

Table S1: Excitation energies, oscillator strength (f), and composition in terms of molecular orbital transitions (only contributions $> 10\%$ included) of the first five excitations of 1T obtained from the linear-response Casida scheme implemented in OCTOPUS using the LDA functional. H stands for HOMO and L for LUMO.

Excitation	Energy (eV)	f	Composition (weight in %)
1	4.59	0.001	H → L+1 (99%)
2	5.59	0.013	H-1 → L (75%); H → L+3 (22%)
3	5.65	0.000	H → L+2 (99%)
4	5.72	0.020	H → L (93%)
5	5.84	0.000	H-1 → L+1 (99%)

Table S2: Excitation energies, oscillator strength (f), and composition in terms of molecular orbital transitions (only contributions $> 10\%$ included) of the first five excitations of 2T obtained from the linear-response Casida scheme implemented in OCTOPUS using the LDA functional. H stands for HOMO and L for LUMO.

Excitation	Energy (eV)	f	Composition (weight in %)
1	3.84	0.107	H → L (95%)
2	4.38	0.000	H-1 → L (55%); H → L+1 (33%)
3	4.62	0.000	H → L+2 (99%)
4	4.66	0.008	H-2 → L (84%)
5	4.69	0.000	H-1 → L (35%); H → L+1 (37%)

Table S3: Excitation energies, oscillator strength (f), and composition in terms of molecular orbital transitions (only contributions $> 10\%$ included) of the first five excitations of 4T obtained from the linear-response Casida scheme implemented in OCTOPUS using the LDA functional. H stands for HOMO and L for LUMO.

Excitation	Energy (eV)	f	Composition (weight in %)
1	2.54	0.283	H → L (96%)
2	2.81	0.000	H-1 → L (36%); H → L+1 (62%)
3	3.58	0.000	H-1 → L (52%); H → L+1 (24%); H-2 → L (15%)
4	3.59	0.010	H-1 → L+1 (13%); H → L+2 (71%)
5	3.75	0.000	H-4 → L (19%); H-2 → L (62%)

Table S4: Excitation energies, oscillator strength (f), and composition in terms of molecular orbital transitions (only contributions > 10% included) of the first five excitations of 6T obtained from the linear-response Casida scheme implemented in OCTOPUS using the LDA functional. H stands for HOMO and L for LUMO.

Excitation	Energy (eV)	f	Composition (weight in %)
1	2.03	0.396	H $\rightarrow$ L (93%)
2	2.14	0.001	H-1 $\rightarrow$ L (45%); H $\rightarrow$ L+1 (54%)
3	2.70	0.038	H $\rightarrow$ L+2 (68%)
4	2.82	0.000	H-1 $\rightarrow$ L (45%); H $\rightarrow$ L+1 (36%)
5	2.85	0.116	H-2 $\rightarrow$ L (40%); H-1 $\rightarrow$ L+1 (51%)

Orbital Contributions to the Population Analysis

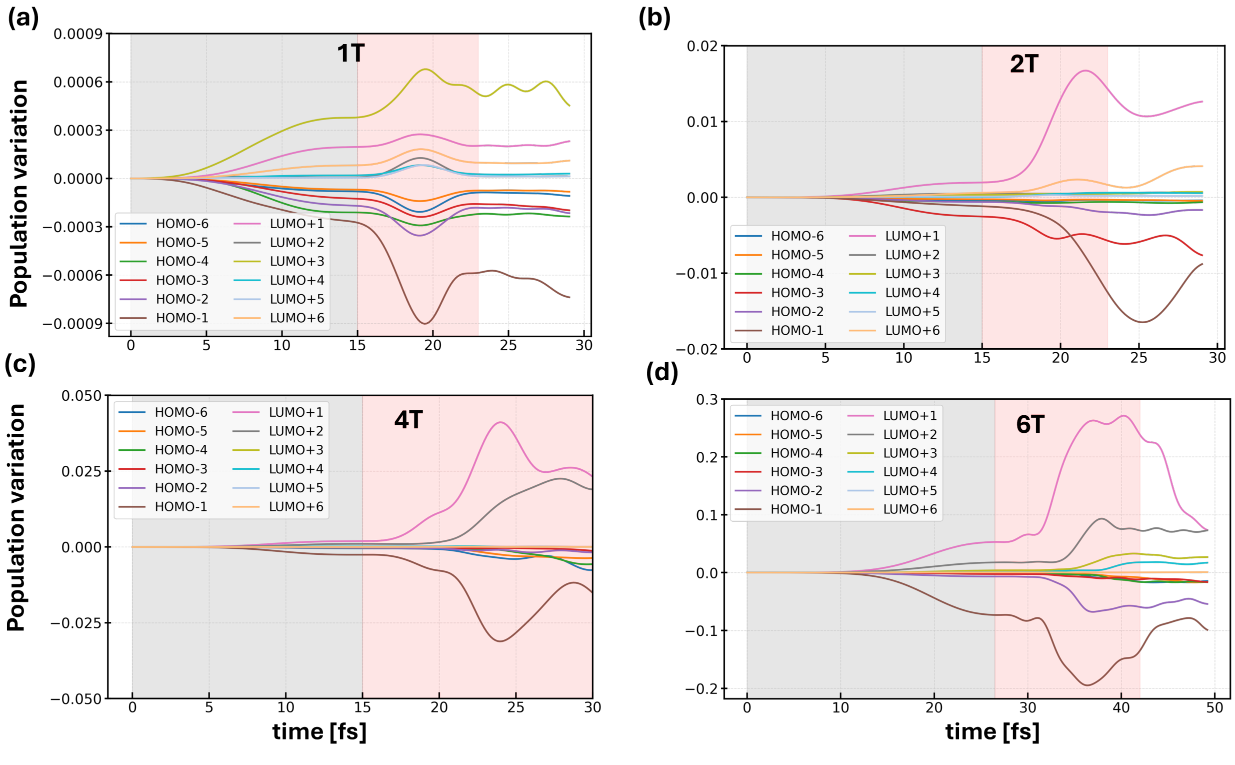

Figure S2: Population dynamics computed in terms of the molecular orbitals of a) 1T, b) 2T, c) 4T, and d) 6T excited by a train of Gaussian pulses in resonance with $S_0 \rightarrow S_1$ (gray area) and with the absorption maximum below the linear onset (red area).

Convergence Tests and Benchmarks

Energy Conservation

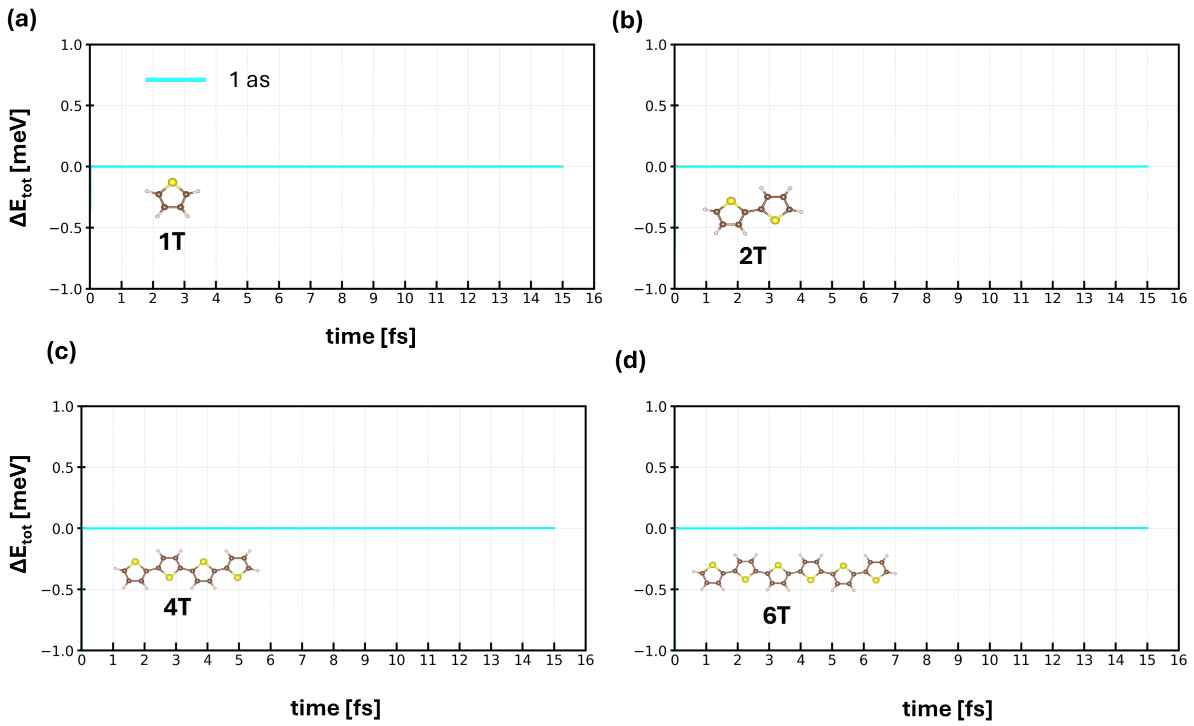

Figure S3: Time evolution of the total energy under an applied kick strength of 0.001 Å^{-1} for a) 1T, b) 2T, c) 4T, and d) 6T. The plots illustrate the variation of the total energy with respect to different time steps throughout the real-time propagation.

We analyzed the variation of the total energy of the molecules during the time propagation, triggered by δ-kick perturbations of different intensities: 0.001 Å^{-1} (Figure S3), 0.05 Å^{-1} (Figure S4), and 1 Å^{-1} (Figure S5). For the weakest perturbation, the energy drift is negligible across all molecules (Figure S3). As the magnitude of the kick and the size of the oligomer increase, deviations in the total energy become larger, up to a few hundred meV for 6T subject to a δ-kick of 0.5 Å^{-1} (Figure S4). For the largest applied kick, $\kappa = 1$ Å^{-1}, the energy drift increases up to the order of 1 eV (Figure S5, cyan curves).

This instability is a known problem related to the time step being insufficient to resolve the highest electronic frequencies generated by strong-field coupling in extended systems.[1] By

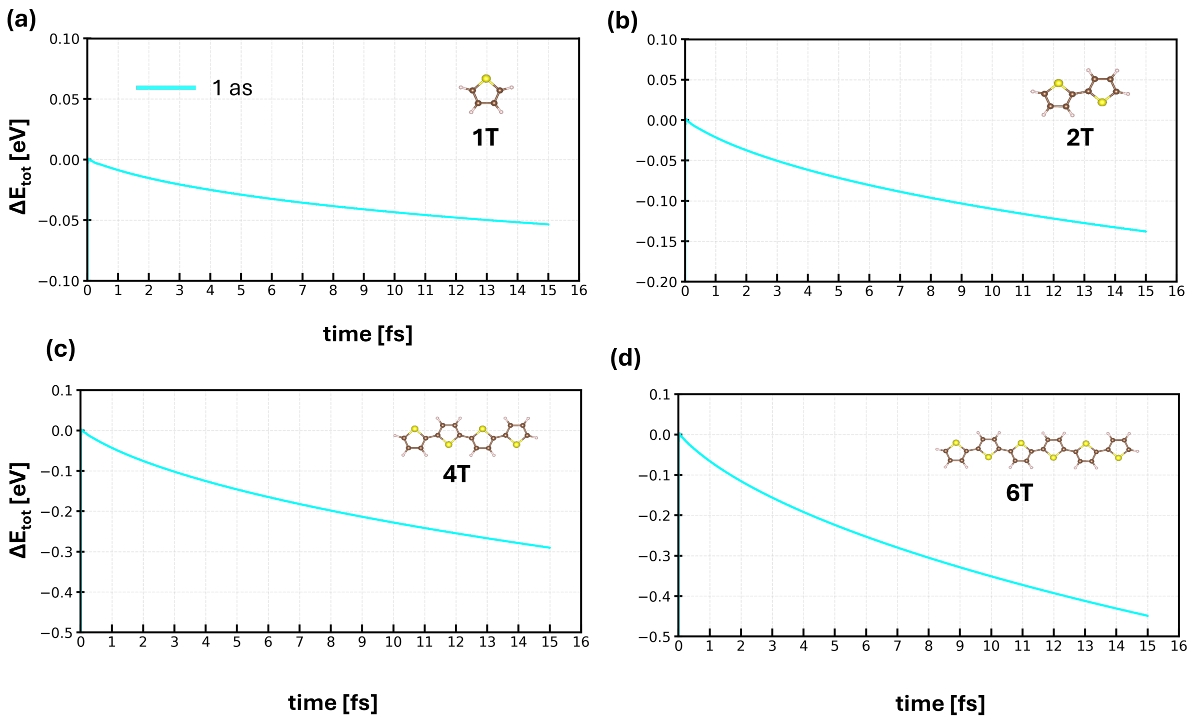

Figure S4: Time evolution of the total energy under an applied kick strength of 0.5 Å^{-1} for a) 1T, b) 2T, c) 4T, and d) 6T. The plots illustrate the variation of the total energy with respect to its initial value throughout the real-time propagation.

systematically reducing the time-step as down to 0.8 as (green curves) and 0.5 as (red curves), the energy drift is effectively reduced by one and two orders of magnitude, respectively (Figure S5).

To conclusively assess the influence of this numerical instability on the calculated physical observables, we compared the nonlinear absorption cross sections triggered by a 1 Å^{-1} δ-kick across the three tested time steps (1 as, 0.8 as, and 0.5 as). As shown in Figure S6, the computed nonlinear cross sections for all considered molecules are identical, regardless of the time step. This result unequivocally confirms that the 1 as time step, despite introducing a non-negligible drift in the total energy, does not generate artifacts in the spectral features that define our nonlinear results. The 1 as time step is thus established as the optimal balance between accuracy of the physical observable and computational efficiency, particularly for large moieties like 6T.

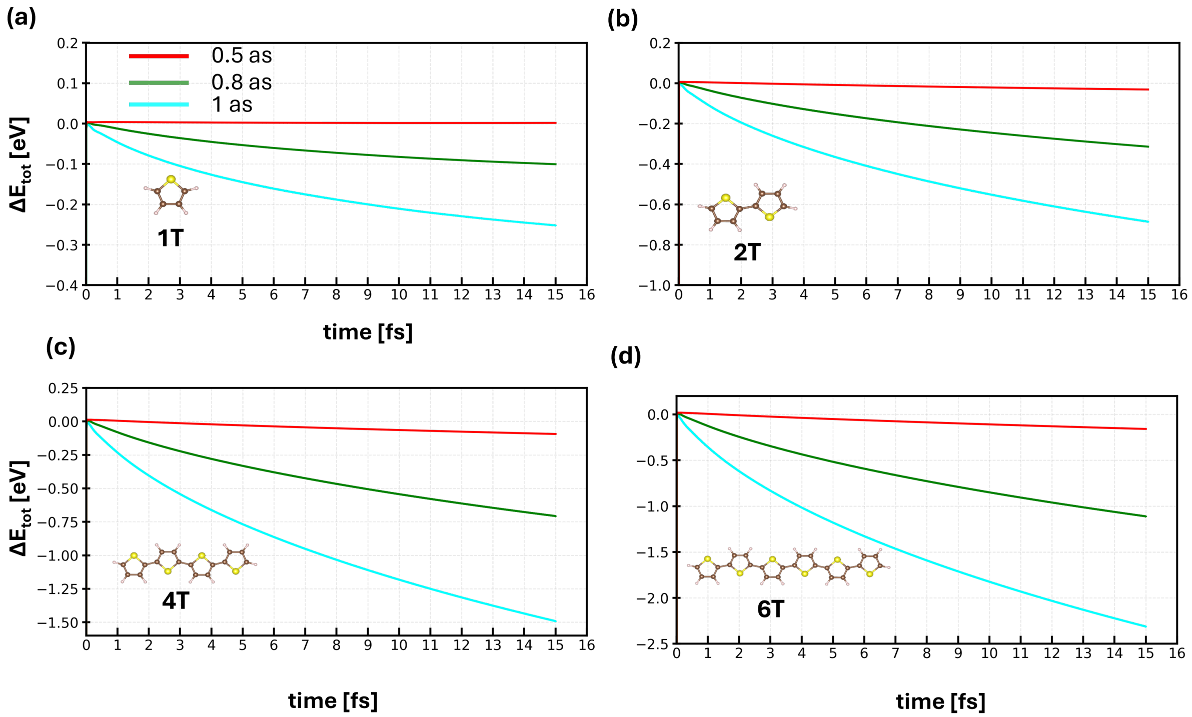

Figure S5: Time evolution of the total energy under an applied kick strength of 1 Å^{-1} for a) 1T, b) 2T, c) 4T, and d) 6T. The plots illustrate the variation of the total energy with respect to its initial value throughout the real-time propagation.

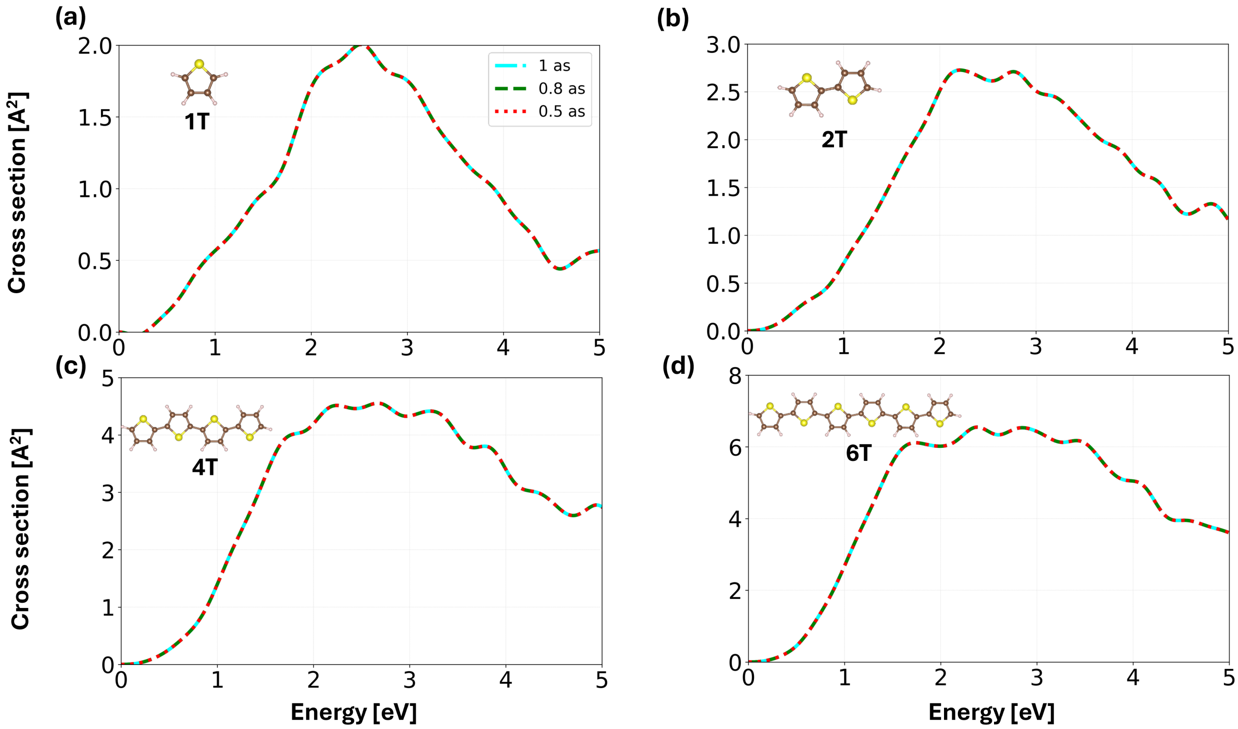

Figure S6: Absorption cross section, calculated as the trace over the three Cartesian components, for (a) 1T, (b) 2T, (c) 4T, and (d) 6T excited by an instantaneous, broadband pulse with a δ-kick intensity of 1 $\mathring{A}^{-1}$, polarized equally along all Cartesian directions using three different time steps: 1 as (cyan), 0.8 as (green), and 0.5 as (red).

Linear Response Calculations

In Tables S5-S12, we report the energy, oscillator strength, and composition of the first 5 excitations of the four considered oligothiophenes computed from linear-response TDDFT using Gaussian16[2] in the adiabatic LDA[3] and with the range-separated hybrid functional CAM-B3LYP.[4] Finally, in Table S13, we compare the energy of the first bright excitation computed for all molecules using LDA (RT-TDDFT and linear response with OCTOPUS using the Perdew-Zunger functional[5] and Gaussian16) and CAM-B3LYP (Gaussian16) with experimental values recorded in solution at room temperature.[6] All theoretical spectra are visualized in Figure S4.

Table S5: Excitation energies, oscillator strength (f) and composition in terms of molecular orbital transitions (only contributions $>10\%$ included) of the first five excitations of 1T obtained from TDDFT calculations with the LDA functional in Gaussian16. H stands for HOMO and L for LUMO.

Excitation	Energy (eV)	f	Composition (weight in %)
1	5.89	0.06	H–1 → L (41%)
2	5.93	0.00	H → L+1 (50%)
3	5.95	0.09	H → L (48%)
4	6.35	0.00	H–1 → L+1 (50%)
5	7.51	0.00	H–2 → L (49%)

Table S6: Excitation energies, oscillator strength (f) and composition in terms of molecular orbital transitions (only contributions $>10\%$ included) of the first five excitations of 1T obtained from TDDFT calculations with the CAM-B3LYP functional in Gaussian16. H stands for HOMO and L for LUMO.

Excitation	Energy (eV)	f	Composition (weight in %)
1	6.10	0.09	H → L (48%)
2	6.19	0.10	H–1 → L (44%)
3	6.54	0.00	H → L+1 (49%)
4	6.56	0.00	H–1 → L+1 (49%)
5	8.07	0.00	H–2 → L (48%)

Table S7: Excitation energies, oscillator strength (f) and composition in terms of molecular orbital transitions (only contributions $> 10\%$ included) of the first five excitations of 2T obtained from TDDFT calculations with the LDA functional in Gaussian16. H stands for HOMO and L for LUMO.

Excitation	Energy (eV)	f	Composition (weight in %)
1	3.87	0.42	H → L (49%)
2	4.49	0.00	H–2 → L (31%); H–1 → L+1 (13%)
3	4.74	0.00	H → L+2 (50%)
4	4.77	0.04	H–3 → L (44%)
5	4.80	0.00	H–4 → L (12%); H–2 → L (15%); H–1 → L+1 (19%)

Table S8: Excitation energies, oscillator strength (f) and composition in terms of molecular orbital transitions (only contributions $> 10\%$ included) of the first five excitations of 2T obtained from TDDFT calculations with the CAM-B3LYP functional in Gaussian16. H stands for HOMO and L for LUMO.

Excitation	Energy (eV)	f	Composition (weight in %)
1	4.35	0.42	H → L (49%)
2	5.47	0.00	H–1 → L (45%)
3	5.69	0.12	H–2 → L (44%)
4	5.77	0.00	H → L+2 (46%)
5	5.80	0.00	H → L+3 (46%)

Table S9: Excitation energies, oscillator strength (f) and composition in terms of molecular orbital transitions (only contributions $> 10\%$ included) of the first five excitations of 4T obtained from TDDFT calculations with the LDA functional in Gaussian16. H stands for HOMO and L for LUMO.

Excitation	Energy (eV)	f	Composition (weight in %)
1	2.96	2.05	H–1 → L+1 (21%); H → L (66%)
2	3.67	0.00	H–1 → L (45%); H → L+1 (51%)
3	4.28	0.00	H–1 → L (49%); H → L+1 (43%)
4	4.31	0.15	H–1 → L+1 (41%); H → L+2 (44%)
5	4.86	0.00	H–2 → L (47%); H → L+2 (42%)

Table S10: Excitation energies, oscillator strength (f) and composition in terms of molecular orbital transitions (only contributions $> 10\%$ included) of the first five excitations of 4T obtained from TDDFT calculations with the CAM-B3LYP functional in Gaussian16. H stands for HOMO and L for LUMO.

Excitation	Energy (eV)	f	Composition (weight in %)
1	3.32	1.21	H → L (48%)
2	4.33	0.00	H–1 → L (16%); H → L+1 (30%)
3	4.79	0.00	H–1 → L (30%); H → L+1 (14%)
4	5.06	0.03	H–5 → L (16%); H → L+2 (14%)
5	5.11	0.00	H–2 → L (35%)

Table S11: Excitation energies, oscillator strength (f) and composition in terms of molecular orbital transitions (only contributions $> 10\%$ included) of the first five excitations of 6T obtained from TDDFT calculations with the LDA functional in Gaussian16. H stands for HOMO and L for LUMO.

Excitation	Energy (eV)	f	Composition (weight in %)
1	2.03	1.64	H → L (49%)
2	2.15	0.00	H–1 → L (24%); H → L+1 (26%)
3	2.76	0.18	H–1 → L+1 (12%); H → L+2 (33%)
4	2.86	0.35	H–2 → L (23%); H–1 → L+1 (23%)
5	2.88	0.00	H–1 → L (23%); H → L+1 (20%)

Table S12: Excitation energies, oscillator strength (f) and composition in terms of molecular orbital transitions (only contributions $> 10\%$ included) of the first five excitations of 6T obtained from TDDFT calculations with the CAM-B3LYP functional in Gaussian16. H stands for HOMO and L for LUMO.

Excitation	Energy (eV)	f	Composition (weight in %)
1	2.96	2.05	H → L (44%)
2	3.67	0.00	H–1 → L (20%); H → L+1 (26%)
3	4.28	0.00	H–1 → L (24%); H → L+1 (19%)
4	4.31	0.15	H–1 → L+1 (17%); H → L+2 (19%)
5	4.86	0.00	H–2 → L (22%); H → L+2 (18%)

Table S13: First excitation energy (in eV) of the oligothiophenes considered in this work computed from RT-TDDFT ($\kappa = 0.001$ Å^{-1}) and from linear-response TDDFT (LR-TDDFT) using the Casida scheme implemented in Octopus with the Perdew-Zunger[5] LDA functional and with Gaussian16 using the LDA and the CAM-B3LYP functional. For 1T, the second excitation is taken for the LDA (Octopus) results, since the first dark one is a numerical artifact. The TDDFT results are compared against experimental absorption data at room temperature in solution (acetonitrile for 1T and dioxane for the other molecules) taken from Ref. 6.

System	RT-TDDFT	LR-TDDFT			Exp. (sol.)
		LDA (Octopus)	LDA (G16)	CAM-B3LYP (G16)	
1T	5.5	5.59	5.89	6.10	5.37
2T	3.8	3.84	3.87	4.35	4.09
4T	2.5	2.54	2.96	3.32	3.16
6T	2.0	2.03	2.03	2.96	2.84

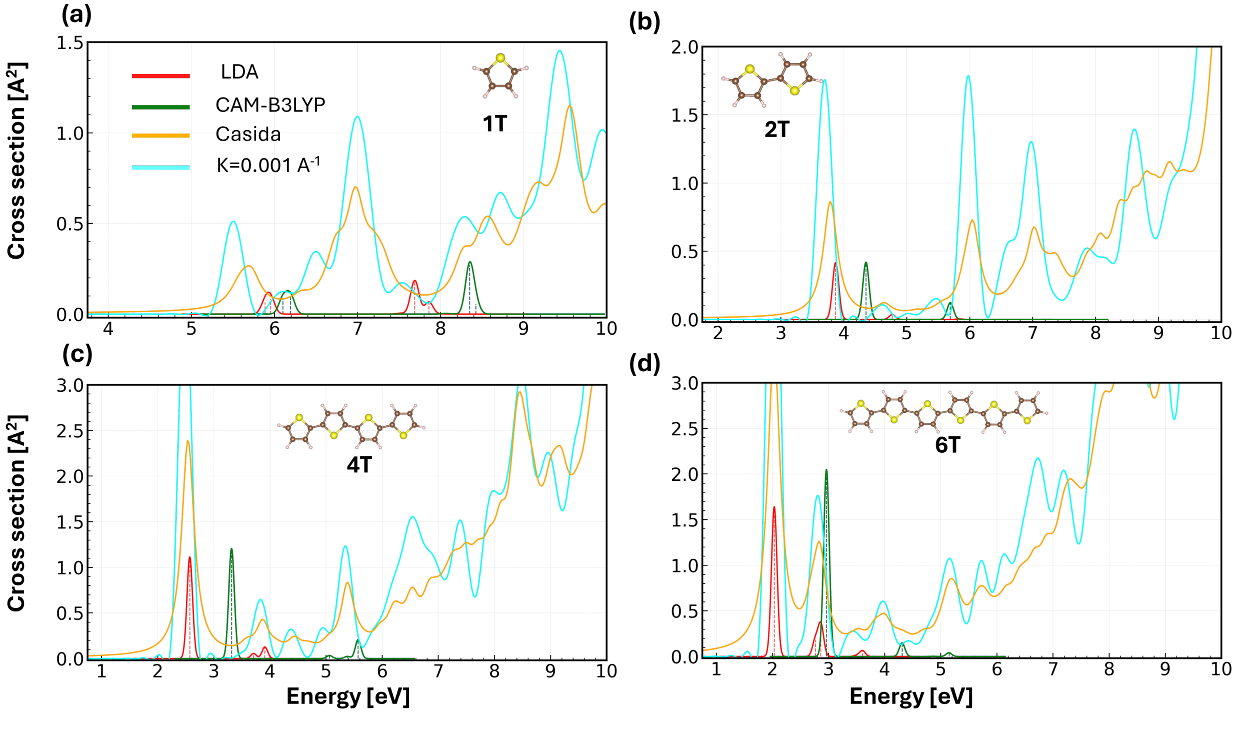

Figure S7: Linear optical absorption spectra of a) 1T, b) 2T, c) 4T, and d) 6T calculated with Gaussian16 using the LDA (red) and CAM-B3LYP functional (green) compared against corresponding results obtained with the Casida scheme in OCTOPUS using LDA (orange). For comparison, the spectra computed from RT-TDDFT results obtained with OCTOPUS using a weak kick of magnitude $\kappa = 0.001$ Å^{-1} using the LDA functional are shown for comparison (cyan).

References

(1) Yost, D. C.; Yao, Y.; Kanai, Y. Propagation of maximally localized Wannier functions in real-time TDDFT. *J. Chem. Phys.* **2019**, *150*, 194113.

(2) Frisch, M. J.; Trucks, G. W.; Schlegel, H. B.; Scuseria, G. E.; Robb, M. A.; Cheeseman, J. R.; Scalmani, G.; Barone, V.; Petersson, G. A.; Nakatsuji, H. et al. Gaussian16 Revision C.01. 2016; Gaussian Inc. Wallingford CT.

(3) Vosko, S. H.; Wilk, L.; Nusair, M. Accurate spin-dependent electron liquid correlation energies for local spin density calculations: a critical analysis. *Can. J. Phys.* **1980**, *58*, 1200–1211.

(4) Yanai, T.; Tew, D. P.; Handy, N. C. A new hybrid exchange–correlation functional using the Coulomb-attenuating method (CAM-B3LYP). *Chem. Phys. Lett.* **2004**, *393*, 51–57.

(5) Perdew, J. P.; Zunger, A. Self-interaction correction to density-functional approximations for many-electron systems. *Phys. Rev. B* **1981**, *23*, 5048–5079.

(6) Becker, R. S.; Seixas de Melo, J.; Macanita, A. L.; Elisei, F. Comprehensive evaluation of the absorption, photophysical, energy transfer, structural, and theoretical properties of α-oligothiophenes with one to seven rings. *J. Phys. Chem.* **1996**, *100*, 18683–18695.
